# Oxidative stress and phosphatidylserine exposure in red cells from patients with sickle cell anaemia

**DOI:** 10.1111/bjh.15441

**Published:** 2018-06-25

**Authors:** Anke Hannemann, David C. Rees, John N. Brewin, Andreas Noe, Ben Low, John S. Gibson

**Affiliations:** ^1^ Department of Veterinary Medicine University of Cambridge Cambridge UK; ^2^ Department of Paediatric Haematology King's College Hospital King's College London School of Medicine London UK

**Keywords:** sickle cell anaemia, oxidants, thiols, calcium, phosphatidylserine exposure

## Abstract

Phosphatidylserine (PS) exposure increases as red cells age, and is an important signal for the removal of senescent cells from the circulation. PS exposure is elevated in red cells from sickle cell anaemia (SCA) patients and is thought to enhance haemolysis and vaso‐occlusion. Although precise conditions leading to its externalisation are unclear, high intracellular Ca^2+^ has been implicated. Red cells from SCA patients are also exposed to an increased oxidative challenge, and we postulated that this stimulates PS exposure, through increased Ca^2+^ levels. We tested four different ways of generating oxidative stress: hypoxanthine and xanthine oxidase, phenazine methosulphate, nitrite and *tert*‐butyl hydroperoxide, together with thiol modification with *N*‐ethylmaleimide (NEM), dithiothreitol and hypochlorous acid (HOCl), in red cells permeabilised to Ca^2+^ using bromo‐A23187. Unexpectedly, our findings showed that the four oxidants significantly reduced Ca^2+^‐induced PS exposure (by 40–60%) with no appreciable effect on Ca^2+^ affinity. By contrast, NEM markedly increased PS exposure (by about 400%) and slightly but significantly increased the affinity for Ca^2+^. Dithiothreitol modestly reduced PS exposure (by 25%) and HOCl had no effect. These findings emphasise the importance of thiol modification for PS exposure in sickle cells but suggest that increased oxidant stress alone is not important.

Sickle cell anaemia (SCA) is one of the commonest severe inherited disorders affecting millions of people worldwide, including some 12 000–15 000 in the UK (Piel *et al*, 2013). Although the aetiology has been known for over 60 years, the condition remains refractory to effective therapy, which is largely supportive. Whilst hydroxycarbamide has been identified as useful for patients with more severe symptoms, its use is not without complications and more effective treatments are keenly sought. Lack of a detailed understanding of the pathogenesis of SCA represents a major impediment to progress. Occlusion of small blood vessels, tissue ischaemia and organ damage, as well as the chronic anaemia, are important features of the condition. Through its pro‐thrombotic and pro‐phagocytic activities, red cell phosphatidylserine (PS) may participate in vascular occlusion and destruction of red cells (Hebbel, 1991; Boas *et al*, 1998; Setty *et al*, 2002; Steffen *et al*, 2011). This paper concerns the mechanism of red cell PS exposure and the role of oxidative damage in sickle cell disease. Understanding this aspect has clear clinical implications, with increased understanding of triggers for the the vaso‐occlusive and haemolytic complications in SCA. In addition, if the mechanisms leading to PS exposure are clarified, findings may suggest potential chemotherapeutic measures to reduce PS exposure and ameliorate some of the clinical complications of SCA.

Phosphatidylserine is usually confined to the inner leaflet of the lipid bilayer in red cell membranes (Kuypers, 1998; Haest, 2003). By analogy to apoptosis in nucleated cells, the process of PS exposure in red cells and their subsequent removal from the circulation by macrophages, has been termed eryptosis (Lang *et al*, 2006). In SCA, a high, but variable proportion of red cells shows PS exposure (Tait & Gibson, 1994; Kuypers *et al*, 1996; de Jong *et al*, 2001; Weiss *et al*, 2012), which further increases upon deoxygenation, haemoglobin polymerisation and the sickling shape change (Lubin *et al*, 1981; Blumenfeld *et al*, 1991; Weiss *et al*, 2012; Cytlak *et al*, 2013). This red cell lipid scrambling may therefore participate in the pathogenesis of SCA, and contribute to both the chronic anaemia and the acute ischaemic episodes characteristic of the condition (Rees *et al*, 2010), and is thus central to the clinical progression of the disease.

In mature red cells, two transport processes predominate in the maintenance of PS asymmetry: an ATP‐dependent aminophospholipid translocase (or “flippase”), which moves PS from the outer to the inner leaflet of the membrane, and a Ca^2+^‐dependent scrambling process (or “scramblase”), which moves PS in either direction (Haest, 2003; Bevers & Williamson, 2010). Rapid PS exposure in mature red cells requires activation of the scramblase together with inhibition of the flippase (Haest, 2003; Barber *et al*, 2009). Elevation of intracellular Ca^2+^ can elicit both these processes (Williamson *et al*, 1992; Basse *et al*, 1996; Woon *et al*, 1999; Haest, 2003; Bevers & Williamson, 2010). Flippase inhibition is generally considered to be more sensitive to Ca^2+^ (Bitbol *et al*, 1987; Basse *et al*, 1996; Kamp *et al*, 2001; Bevers & Williamson, 2010) although more recent work suggests that, in fact, lipid scrambling is activated at similar Ca^2+^ concentrations to flippase inhibition (Weiss *et al*, 2012; Cytlak *et al*, 2013). Nevertheless, our understanding of the conditions and mechanisms of PS exposure in red cells from SCA patients remains incomplete.

Other mechanisms may also participate in PS exposure. Thus, recently, the expulsion of membrane‐bound organelles by autophagy during maturation of erythrocyte precursors has been shown to result in punctuate externalised PS localised to discrete patches (Mankelow *et al*, 2015). Furthermore, PS may be expelled as microvesicles. The involvement of these particles in the pathogenesis of SCA and other conditions has been widely postulated (Piccin *et al*, 2007, 2015a, 2017; Nebor *et al*, 2014).

Red cells from patients with SCA contain the abnormal haemoglobin, HbS, which is able to polymerise upon deoxygenation and thereby cause cell sickling. The subsequent alterations in red cell morphology and rheology have critical deleterious consequences for red cell function. Sickle red cells also have an abnormally high cation permeability involving three main transport processes (Lew & Bookchin, 2005): the KCl cotransporter, the Ca^2+^‐activated K^+^ channel (or Gárdos channel) and an ill‐defined cation conductance (sometimes termed P_sickle_ – Lew *et al*, 1997). These transporters combine to mediate solute loss with water following osmotically. The consequent elevation of red cell HbS concentration, [HbS], has the effect of markedly reducing the lag time to HbS polymerisation following deoxygenation (Eaton & Hofrichter, 1987). P_sickle_ is activated by deoxygenation, HbS polymerisation and red cell shape change (Mohandas *et al*, 1986). P_sickle_‐mediated Ca^2+^ entry has an established role in activation of the Gárdos channel and may therefore increase dehydration (Rhoda *et al*, 1990; Lew *et al*, 1997). The rise in intracellular Ca^2+^, [Ca^2+^], may also play a role in lipid scrambling. Previous findings are consistent with deoxygenation‐induced Ca^2+^ entry, predominantly via P_sickle_, contributing to elevation of intracellular Ca^2+^ levels and leading to increased PS exposure (Weiss *et al*, 2012; Cytlak *et al*, 2013). Low micromolar levels of Ca^2+^ are required (Weiss *et al*, 2012; Cytlak *et al*, 2013), which although representing a higher Ca^2+^ affinity for scrambling than hitherto thought, still represent relatively high levels, even for deoxygenated sickle cells (Etzion *et al*, 1993).

Oxidative damage has long been associated with PS exposure in red cells (Jain & Shohet, 1984; Jain & Williams, 1985; Kuypers, 1998; Lang *et al*, 2002, 2012). This is particularly significant in diseases such as SCA in which vascular oxidative stress is increased (Rice‐Evans *et al*, 1986) with accumulations of highly reactive oxygen species (ROS) including the superoxide anion, hydrogen peroxide and the hydroxyl radical (Sies, 1997; Chirico & Pialoux, 2012; Voskou *et al*, 2015). Myeloperoxidase released from activated neutrophils may also add to oxidant challenge in SCA, through production of hypochlorous acid (HOCl) from hydrogen peroxide (Vissers *et al*, 1994; Mutze *et al*, 2003; Zhang *et al*, 2013). Thiol oxidation has also been associated with both inhibition of the flippase and stimulation of the scramblase (Morrot *et al*, 1989; Connor & Schroit, 1990; Devaux & Zachowski, 1994; Martin & Jesty, 1995; de Jong & Kuypers, 2006). In addition, the normal antioxidant capacity of the red cell is compromised in SCA patients, with low availability of antioxidant enzymes, reduced levels of glutathione (GSH) and also of non‐enzymatic antioxidants, such as vitamins C and E (Silva *et al*, 2013). Oxidative damage has also been associated with deleterious effects on red cell cation balance. It causes inhibition of the plasma membrane Ca^2+^ pump (Shalev *et al*, 1981; Zaidi *et al*, 2003), which maintains intracellular Ca^2+^ concentrations at low levels, perhaps via interaction with calmodulin (Gao *et al*, 2001), and also has marked effects on red cell potassium permeability (Gibson & Muzyamba, 2003a,2003b). A randomized controlled trial has also recently shown that l‐glutamine, believed to act predominantly as an antioxidant, reduced the frequency of acute pain in SCA (Niihara *et al*, 2016). Taken together, these observations are indicative of an important role of oxidative stress in pathogenesis of SCA.

We therefore postulated that oxidative challenge in SCA patients may increase red cell PS scrambling and alter the Ca^2+^ affinity of the process. We investigated the interaction between Ca^2+^ and oxidant challenge in red cells from SCA patients. Oxidative stress was provided by extracellular and intracellular superoxide anion generating systems, nitrite and *tert*‐butyl hydroperoxide. The effect of thiol modifications were also investigated using *N*‐ethylmaleimide (NEM), dithiothreitol (DTT) and HOCl.

## Materials and methods

### Sample collection and handling

The study was approved by the National Research Ethics Committee (reference 16/LO/1309). All research was conducted with ethical approval and in accordance with the Helsinki Declaration of 1975, as revised in 2008. Consented blood samples were taken from children homozygous for SCA (HbSS), into EDTA. The study involved 37 patients (23 males) with an average age of 11·3 ± 3·8 years. Nineteen patients were receiving hydroxycarbamide therapy and eighteen were not. Mean % HbF levels and reticulocyte counts were 10·2 ± 5·1 and 13·2 ± 5·1, respectively (all means ± standard deviation [SD], *n* = 37).

### Chemicals, solutions, red cell preparation and full methology

The following is a brief description of the methods used. Full details of chemicals used, solutions, red cell preparation and methology are given in the Appendix S1.

### Oxidant challenge and thiol modifications

Extracellular superoxide anion (SOA) and hydrogen peroxide were generated by incubation with mixtures of hypoxanthine (HO; 2 mmol/l) and xanthine oxidase (XO) at concentrations up to 0·1 U/ml, a manoeuvre that is well established to provide an oxidative challenge to red cells (Baskurt *et al*, 1998; Rogers *et al*, 2009). Using data from pilot experiments, most work was carried out with a [XO] of 0·015 U/ml. Phenazine methosulphate (PMS, 0·01–0·4 mmol/l) was used to generate intracellular superoxide anion. Nitrite (NO_2_) (to generate methaemoglobin) and *tert*‐butyl hydroperoxide (*t*BHP) (to generate peroxyl and alkoxyl derivatives) were used at concentrations of 1–20 mmol/l and 0·05–1·0 mmol/l, respectively. HOCl was prepared immediately before experiments and used at a final concentration of 0·001–1 mmol/l. Red cells were incubated with oxidants for 30 min at 37°C prior to measuring PS. The thiol modifiers NEM and DTT were used at concentrations of 1 mmol/l and 0·25 mmol/l and red cells were pre‐incubated for 30 min at 37°C, followed by 30 min at 37°C without or with oxidants. DTT was excluded from the second incubation step to prevent red cell lysis.

### Measurement of red cell oxidative stress and red cell morphology

To measure intracellular oxidative load, red cells were first loaded with CM‐H_2_DCF‐DA (100 μmol/l) in the dark (for 30 min at 37°C) and washed twice. On permeation into red cells, this fluorophore is hydrolysed to the non‐fluorescent di‐hydro compound which, in the presence of reactive oxygen species (ROS), is oxidized to highly fluorescent CM‐H_2_dichlorofluorescein (CM‐H_2_‐DCF). CM‐H_2_DCF fluorescence was measured in the FL1 channel of a BD Accuri C6 flow cytometer (Becton Dickinson, Oxford, UK). Median FL1 fluorescence was used as an indication of the magnitude of intracellular oxidative load. For each fluorescence measurement, 10 000 events were gated. To examine red cell morphology, cells were fixed through addition of glutaraldehyde (0·3%) and examined under light microscopy, typically examining several hundred cells.

### Measurement of externalised PS using fluorescent lactadherin fluorescein isothiocynate (LA‐FITC)

Accessible PS was labelled with the fluorescent PS marker, LA‐FITC. Usually this marker can only gain access to exposed PS on the outer leaflet of the membrane bilayer, unless membrane integrity has been compromised. To promote lipid scrambling, red cells were incubated with the Ca^2+^ ionophore bromo‐A23187 (6 μmol/l) to permeabilise red cells to Ca^2+^ at various [Ca^2+^]_o_s clamped using EDTA (2 mmol/l). LA‐FITC was detected in the FL1 channel of a BD Accuri C6 flow cytometer using logarithmic gain (as for CM‐H_2_‐DCF fluorescence). For each measurement, 10 000 events were gated. XO, PMS and *t*BHP all showed various degrees of autofluorescence in unlabelled red cells in the absence of any fluorophore. It was therefore critical to choose appropriate concentrations of these oxidants that elevated intracellular ROS levels whilst keeping autofluorescence to manageable values. In all cases, compensation for the fluorescent overspill was carefully set using oxidant‐treated red cells, unlabelled with LA‐FITC. NO_2_ showed no significant autofluorescence in the FL1 channel.

### Measurement of externalised PS using a prothrombinase assay

Phosphatidylserine exposure was also assessed by generating thrombin using a prothrombinase assay, following the method of Bevers *et al* (1982). Factor Va and Factor Xa (0·5 and 0·25 U/ml final concentration, respectively) were added, and after 2 min thrombin formation was initiated by adding prothrombin (1·1 μmol/l final concentration). After 2 and 5 min, red cell aliquots were removed and thrombin formation stopped by the addition of EDTA. Thrombin levels were then measured by adding the chromogenic substrate ThrombinChrom (0·125 mmol/l final concentration) with absorbance measured at 415 nm in an iMark microplate reader (Bio‐Rad, Hemel Hempstead, UK). In intact red cells, this thrombin assay measures only externalised PS. Additional aliquots of red cells were also lysed hypotonically through the addition of water, after which the prothrombinase assay was repeated. In lysed red cells, thrombin formation will be initiated by PS on both the inner leaflet of the membrane as well as externalised PS and thus gives a measure of total red cell PS. The assay will therefore indicate whether PS has been lost from the red cells, for example as microvesicles. Although more complicated to carry out, this prothrombinase assay has the advantage of being immune to any problems with autofluorescence or setting of positive gates in the flow cytometer.

### Measurement of red cell membrane integrity

This assay used a fluorescently labelled immunoglobulin against Hb (Alexa Fluor 647 measured in FL4 of FACS) to indicate membrane integrity. Immunoglobin (molecular weight 15 kDa) cannot cross the red cell membrane unless its permeability barrier has been compromised, therefore labelling was taken as an indication of loss of membrane integrity. If this was the case, LA‐FITC would also be expected to gain access to the red cell interior (as well as the exterior) and therefore label PS in both the inner, as well as the outer, bilayer of the membrane. Positive LA‐FITC red cells would therefore not be restricted to those with only externalised PS. Red cells were treated with *t*BHP (0–1 mmol/l) in high potassium HEPES‐buffered saline (HK‐HBS; pH 7·4) for 30 min at 37°C), washed and resuspended. Red cells were then labelled for anti‐Hb, PS and ROS, separately or in paired assays. Loss of CM‐H_2_DCF signal or increase in PS signal concomitant with increased anti‐Hb signal would indicate that cell integrity was compromised. Four aliquots were prepared: (i) CM‐H_2_DCF‐labelled only, (ii) LA‐FITC‐labelled only, (iii) dual labelled with CM‐H_2_DCF and Alexa Fluor 647 conjugated anti‐Hb subunit α immunoglobulin (1:100 dilution) and (iv) dual labelled with LA‐FITC and Alexa Fluor 647 conjugated anti‐Hb subunit α immunoglobulin (1:100 dilution). Red cells were pelleted, washed once, resuspended and kept on ice in the dark until flow cytometry analysis. Median CM‐H_2_DCF and LA‐FITC fluorescence were detected as described above. Median fluorescence of Alexa Fluor 647 conjugated anti‐Hb was detected in the FL4 channel of a BD Accuri C6 flow cytometer.

### Statistics

Results are presented as means ± SD or standard error of the mean (SEM) for blood samples from *n* different individuals. Where appropriate, comparisons were made using 2‐tailed Student's *t*‐tests and *P* < 0·05 was considered as significant.

## Results

### The effect of oxidant stress using xanthine oxidase/hypoxanthine mixtures, PMS and nitrite

Initially, three oxidants were selected to represent a range of different oxidant challenges: XO/HO (2 mmol/l) mixtures to generate extracellular ROS including SOA and hydrogen peroxide (Baskurt *et al*, 1998; Rogers *et al*, 2009), PMS to generate SOA intracellularly (Nishikimi *et al*, 1972; Maridonneau *et al*, 1983) and NO_2_ which promotes formation of methaemoglobin (metHb) (Muzyamba *et al*, 2000).

**Figure 1:**
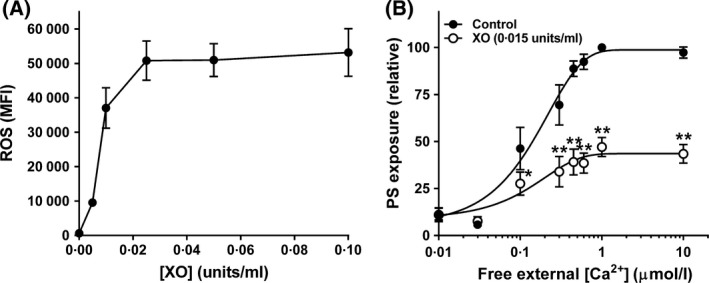
Effect of xanthine oxidase and hypoxanthine (XO/HO) mixtures on accumulation of reactive oxygen species (ROS) and phosphatidylserine (PS) exposure in red cells from patients with sickle cell anaemia (SCA). (A) Red cells were pre‐loaded with CM‐H_2_DCF‐DA (100 μmol/l) to measure ROS levels [as median fluorescence intensity (MFI)] or treated with the same final concentration of dimethyl sulphoxide [DMSO] (control) before incubation with hypoxanthine (HO, 2 mmol/l) and xanthine oxidase (XO, 0–0·1 U/ml) mixtures for 30 min at 37°C (*n* = 2). (B) Red cells were permeabilised to Ca^2+^ with the ionophore bromo‐A23187 (6 μmol/l), and incubated in different free [Ca^2+^]_o_s, maintained using Ca^2+^ / 2 mmol/l EGTA mixtures, at 0·5% haematocrit for 30 min at 37°C in the absence (filled circles) or presence (open circles) of HO (2 mmol/l)/XO (0·015 U/ml) mixtures after which externalised PS was labelled with LA‐FITC, *n* = 8. PS exposure was normalised to that of control red cells at 1 μmol/l free [Ca^2+^]_o_ (31·1 ± 3·9% of total red cells). Symbols represent means ± SEM for red cells from *n* different individuals. *P < 0·05; **P < 0·005.

Control experiments were carried out to establish the appropriate concentrations of oxidants for use in subsequent work using the fluorophore CM‐H_2_DCF to measure intracellular red cell redox state. With XO/HO mixtures, CM‐H_2_DCF fluorescence increased with increasing enzyme activity until it reached a plateau at 0·025 units XO/ml (Fig 1A). CM‐H_2_DCF fluorescence also showed a similar response for PMS and NO_2_, with plateaux fluorescence (arbitrary units) of 78 690 ± 1423 (*n* = 4) achieved at 0·1 mmol/l for PMS and of 89 325 ± 4876 (*n* = 5) at 10 mmol/l for NO_2_, and concentrations of about 0·05 mmol/l for PMS and 6 mmol/l for NO_2_ required half maximal CM‐H_2_DCF fluorescence. The levels of oxidants therefore chosen for further work were 0·015 U/ml, 0·1 mmol/l and 10 mmol/l for XO, PMS and NO_2_, respectively.

### The effect of XO/HO, PMS and NO_2_ on PS exposure using fluorescein isothiocyanate‐conjugated lactadherin (LA‐FITC)

Phosphatidylserine was first measured by flow cytometry using LA‐FITC. If the red cell membrane remains intact, LA‐FITC will only have access to externalised PS. Red cells were loaded intracellularly with Ca^2+^ using the ionophore bromo‐A23187 (6 μmol/l) and extracellular [Ca^2+^], [Ca^2+^]o, from 0 to 10 μmol/l, clamped using appropriate Ca^2+^ / EGTA (2 mmol/l) mixtures. Under these conditions, because of the Donnan ratio, free intracellular [Ca^2+^], [Ca^2+^]_i_ is about double that of free extracellular values (Flatman, 1980; Muzyamba *et al*, 2006). Previous experiments have shown that PS exposure in Ca^2+^ permeabilised red cells occurs when free [Ca^2+^]_o_ is increased above about 0·1 μmol/l (Weiss *et al*, 2012; Cytlak *et al*, 2013). Similar findings were found in the present work with half maximal PS exposure observed at a free [Ca^2+^]_o_ of 0·34 ± 0·02 μmol/l (*n* = 22). When red cells were simultaneously exposed to any of the first three oxidants (XO/HO mixtures, PMS or NO_2_), PS exposure was unaffected in the absence of Ca^2+^ loading but significantly reduced in its presence – to 47 ± 5% (*n* = 8, *P* < 0·005) of controls for XO/HO mixtures, 43 ± 6% (*n* = 7, *P* < 0·005) for PMS and 64 ± 7% (*n* = 9, *P* < 0·05) for NO_2_ (all means ± SEM). This effect is shown for XO/HO mixtures in Fig 1B. There were no obvious differences in the response of red cells from SCA patients treated with hydroxycarbamide, with PS exposure being reduced in the presence of oxidants by about 50% whether or not patients were treated with this drug. The effect of oxidants was also similar in red cells from normal (HbAA) individuals. For example, PMS reduced PS exposure to 43 ± 18% (*n* = 3) of control values in HbAA red cells.

### The effect of XO/HO, PMS and NO_2_ on PS exposure using prothrombinase assays

The decrease in PS exposure in the presence of these oxidants was unexpected as oxidants are generally thought to increase PS exposure in a number of cell types, including red cells from normal individuals and SCA patients (Cimen, 2008; Mohanty *et al*, 2014; Voskou *et al*, 2015). A possible explanation lies in the ability of red cells to shed PS into the incubation media either as the free lipid or in microvesicles (Piccin *et al*, 2015b), which may reduce the remaining PS present on the outside of the red cell membrane despite an increase in scrambling. To test for this possibility and to gain estimates of total red cell PS levels (i.e. that present in both bilayers of the membrane) and thereby exclude possible differential PS shedding, some of the above experiments were repeated using a prothrombinase assay to quantitate for PS. Prothrombinase assays also have the advantage that they exclude any potential artefact through the ability of many oxidants to cause autofluorescence interfering with flow cytometry measurements.

**Figure 2:**
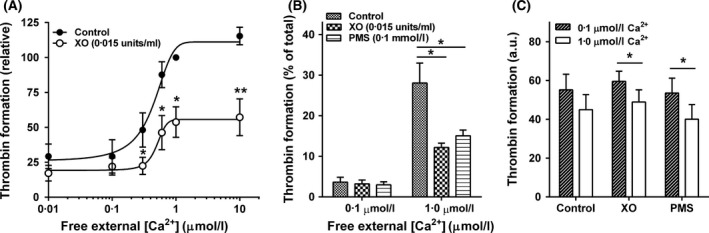
Prothrombinase activity in intact (A and B) or hypotonically lysed (C) red cells from patients with sickle cell anaemia. Red cells were treated as in Fig 1B with thrombin formation used as a measure of accessible phosphatidylserine (PS). (A) Thrombin formation in the absence (filled circles) or presence (open circles) of HO (2 mmol/l)/XO (0·015 U/ml) mixtures over a range of free extra cellular [Ca^2+^] ([Ca^2+^]_o_; 0·1–10 μmol/l), with thrombin formation per min normalised to that of control red cells at 1 μmol/l free [Ca^2+^]_o_. (B) Thrombin formation in the absence (control) or presence of HO (2 mmol/l)/XO (0·015 U/ml) mixtures or PMS (0·1 mmol/l), given as a percentage relative to the total value in lysed red cells at two different free [Ca^2+^]_o_s (0·1 and 1 μmol/l), indicative of prothrombin activity due to externalised PS present on only the outer bilayer of the red cell membrane. (C) Total thrombin formation at 0·1 and 1 μmol/l free [Ca^2+^]_o_ in the absence and presence of HO (2 mmol/l)/XO (0·015 U/ml) mixtures or PMS (0·1 mmol/l) as measured by thrombin formation (in arbitrary units, AU) of hypotonically lysed red cells to give prothrombin activity of total PS present on both the inner and outer bilayers of the red cell membrane. Symbols and histograms represent means ± SEM for red cells from 4 to 5 different individuals. *P < 0·05; **P < 0·005.

Intact red cells (i.e. in which prothrombinase has access only to externalised PS) were loaded with Ca^2+^ (using bromo‐A23187 as above). PS levels using the prothrombinase assay gave the same Ca^2+^ dependence as that for LA‐FITC‐labelled PS (Fig 2A), with half maximal activity achieved at a free [Ca^2+^]_o_ of 0·6 ± 0·1 μmol/l. Treatment with XO/HO mixtures or PMS again inhibited PS levels by about 50% in the prothrombinase assay (Fig 2A for XO/HO mixtures over a full Ca^2+^ titration; Fig 2B for both XO/HO mixtures and PMS at two free [Ca^2+^]_o_s of 0·1 and 1·0 μmol/l). These levels of inhibition were similar level to that observed in flow cytometry experiments using LA‐FITC to label exposed PS.

Phosphatidylserine levels were also measured in hypotonically lysed red cells using the prothrombinase assay. Hypotonic lysis allows access of the assay to PS in both bilayers of the red cell membrane and is thus a measure of total red cell PS. Under these conditions, total thrombin formation did not vary at either 0·1 or 1 μmol/l free [Ca^2+^]_o_ when cells were compared in the absence or presence of XO/HO mixtures or PMS (Fig 2C). There was a loss of about 20% of total PS when free [Ca^2+^]_o_ was raised from 0·1 to 1 μmol/l (Fig 2C), consistent with Ca^2+^‐induced PS shedding, but this reduction was similar in extent in the absence or presence of either oxidant.

From these observations, it is unlikely that the observed reduction in PS measurements in the presence of oxidants was due to an overall loss of PS through oxidant‐induced shedding into the extracellular media, excluding red cells as a possible source of PS‐containing microvesicles in response to oxidant challenge (Piccin *et al*, 2007; Piccin *et al*, 2015a) although they may participate in Ca^2+^‐induced PS loss.

### The effect of tBHP on PS exposure

**Figure 3:**
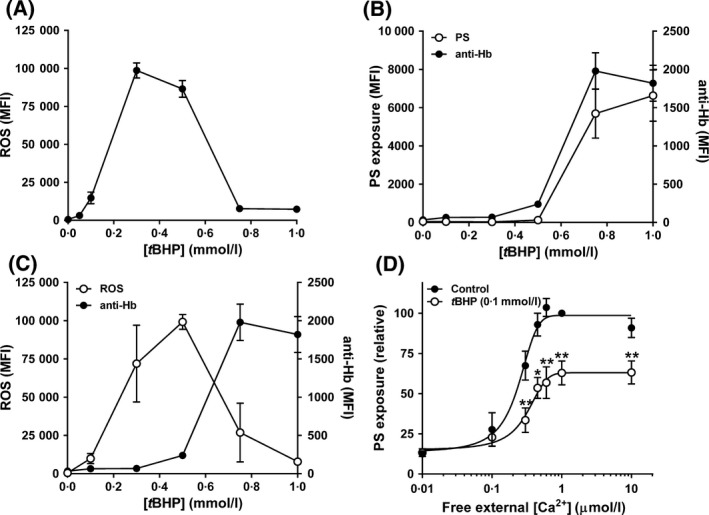
Effect of *tert*‐butyl hydroperoxide (*t*BHP) on accumulation of reactive oxygen species (ROS), phosphatidylserine (PS) exposure and membrane integrity in red cells from patients with sickle cell anaemia. (A) Red cells were pre‐loaded with CM‐H_2_DCF‐DA (100 μmol/l) to measure ROS levels or treated with the same final concentration of dimethyl sulphoxide [DMSO] (control) before incubation with *t*BHP (0–1 mmol/l) at 0·5% haematocrit for 30 min at 37°C in HK‐HBS (*n* = 4). (B) Red cells were double labelled with CM‐H_2_DCF to measure ROS levels (left ordinate) and with Alexa Fluor 647 anti‐Hb α chain (labelled anti‐Hb; right ordinate) to correlate ROS levels with labelling of intracellular haemoglobin (*n* = 3). (C) Red cells were double labelled with LA‐FITC to measure accessible PS and (left ordinate) and with Alexa Fluor 647 anti‐Hb α chain (labelled anti‐Hb; right ordinate) to correlate PS labelling with that of intracellular haemoglobin (*n* = 3). (D) Red cells were permeabilised to Ca^2+^ as in Fig 1B at 0·5% haematocrit for 30 min at 37°C in the absence (filled circles) or presence (open circles) of *t*BHP (0·1 mmol/l) after which externalised PS was labelled with LA‐FITC. PS exposure was normalised to that of control red cells at 1 μmol/l free [Ca^2+^]_o_ (29·3 ± 3·4% of total red cells, *n* = 7). Symbols represent means ± SEM for red cells from *n* different individuals. *P < 0·05; **P < 0·005.

A fourth oxidant, *t*BHP, generates peroxyl and alkoxyl derivatives (Davies, 1989) and has previously been reported to increase PS exposure in red cells from SCA patients – using a concentration of up to 1 mmol/l in a 2‐h incubation (Lang *et al*, 2002). This oxidant behaved differently to the previous three. CM‐H_2_DCF fluorescence, indicative of oxidative load, increased with the concentration of *t*BHP until 0·3 mmol/l, but thereafter, at higher concentrations, fluorescence decreased markedly such that, at 0·75 mmol/l *t*BHP levels had fallen almost to control values (Fig 3A). With [*t*BHP]s of ≥0·5 mmol/l, when tested with LA‐FITC, the percentage of red cells positive for LA‐FITC fluorescence did indeed increase (from 1% to 86 ± 2% at 1 mmol/l *t*BHP) to include most of the red cell population (Fig 3B).

At high [*t*BHP] red cells become markedly leaky to cations (Ney *et al*, 1990). We postulated, therefore, that concentrations of *t*BHP higher than about 0·3 mmol/l may cause disintegrity of the red cell membrane and thereby account for the fall in CM‐H_2_DCF fluorescence. To test this hypothesis, cells were incubated with Alexa Fluor 647‐labelled anti‐Hb α chain immunoglobulin as well as with CM‐H_2_DCF‐AM and LA‐FITC. At higher levels of *t*BHP, CM‐H_2_DCF fluorescence again declined in an increasingly large population of red cells whilst the same red cells simultaneously became positive for Alexa Fluor 647 fluorescence, indicative of entry of anti‐Hb α chain immunoglobulin into the red cells (Fig 3C). At these higher [*t*BHP], PS positive red cells were also positive for anti‐Hb fluorescence (Fig 3C). That a large immunoglobulin (15 kDa) could gain access to the inside of the red cell implies the presence of substantial holes in the membrane that could result in the loss of red cell contents or of CM‐H_2_DCF itself, accounting for the fall in CM‐H_2_DCF fluorescence whilst access of LA‐FITC to the interior of the red cell would label PS remaining on the inside of the membrane as well as externalised PS. When *t*BHP concentrations were used at which red cells became positive for CM‐H_2_DCF fluorescence whilst remaining negative for Alexa Fluor 647 fluorescence, exposed PS labelled with LA‐FITC decreased in the presence of *t*BHP to 63 ± 8% (Fig 3D; means ± SEM, *n* = 7, *P* < 0·005) of controls, to a similar extent to that observed for XO/HO mixtures, PMS and NO_2_.

The presence of *t*BHP also increased cell granularity, as measured by higher side scatter (SSC) values. Compared to controls, SSC was already increased significantly at 0·3 mmol/l *t*BHP (159 ± 10%, *P* < 0·001, *n* = 6), reaching 211 ± 16% of control levels at 1 mmol/l *t*BHP (*P* < 0·001, *n* = 6). FSC also increased but changes were more variable, with a small increase to 112 ± 3% of control levels at 1 mmol/l *t*BHP (*P* < 0·005, *n* = 6). By contrast, SSC and FSC varied by less than 5% when cells were exposed to either PMS or NO_2_. Although changes were more pronounced when using mixtures of HO and higher concentrations of XO (0·1 U/ml), they were not significant (FSC: 108 ± 4%, SSC: 116 ± 11%). Notwithstanding, when observed under light microscopy (20× magnification), there was no obvious difference in morphology between untreated red cells and those exposed to any oxidant, including *t*BHP (up to 1 mmol/l).

### The effect of sulphydryl‐modifying compounds: NEM, DTT and HOCl

**Figure 4:**
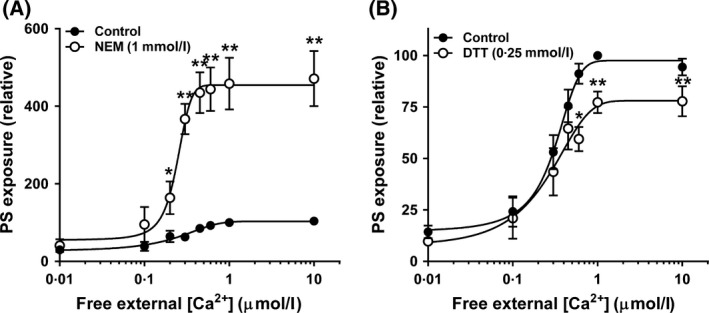
Effect of thiol modification on phosphatidylserine (PS) exposure of red cells from sickle cell anaemia patients. Red cells were pre‐incubated at 4% haematocrit in the absence (solid circles) or presence (open circles) of *N*‐ethylmaleimide (NEM) and dithiothreitol (DTT), then permeabilised to Ca^2+^ as in Fig 1B for 30 min at 37°C, after which accessible PS was labelled with LA‐FITC. (A) Effect of NEM (1 mmol/l) on PS exposure (*n* = 8); (B) Effect of DTT (0·25 mmol/l) on PS exposure (*n* = 10). PS exposure was normalised to that of control red cells (in the absence of thiol modifiers) at 1 μmol/l free [Ca^2+^]_o_ (NEM control: 21·2 ± 4·7%, DTT control: 24·3 ± 4·6% of total red cells). Symbols represent means ± SEM for red cells from *n* different individuals. *P < 0·05; **P < 0·005.

Exposure of red cells to the sulphydryl oxidising reagent NEM (1 mmol/l) has been shown to stimulate PS exposure in human and mouse red cells through inhibiting the flippase and activating the scramblase (Connor & Schroit, 1990; Martin & Jesty, 1995; Kamp *et al*, 2001; de Jong & Kuypers, 2006). This compound was tested here in Ca^2+^‐clamped red cells from SCA patients. PS exposure was increased markedly in the presence of NEM (1 mmol/l), by about 400%, with the Ca^2+^ affinity of scramblase increasing from a 50% maximal response (EC_50_) value of 0·4 ± 0·07 to 0·26 ± 0·03 μmol/l free [Ca^2+^]_o_ (*n* = 6, *P* < 0·03) (Fig 4A). Interestingly, the CMH_2_DCF signal in the presence of 1 mmol/l NEM increased by only 2‐fold compared to that in untreated red cells, to a much lower level of that obtained with the other oxidant, showing that this thiol reagent had little effect on intracellular ROS levels. In the presence of DTT (0·25 mmol/l), which protects thiol groups from oxidation, PS exposure was reduced by about 25% (Fig 4B), with no change in Ca^2+^ dependence of the scrambling process.

**Figure 5:**
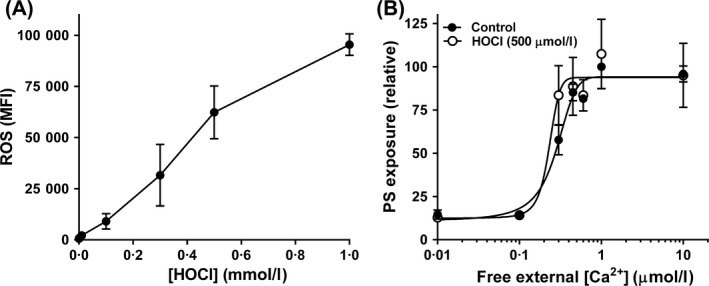
Effect of hypochlorous acid (HOCl) on phosphatidylserine (PS) exposure of red cells from sickle cell anaemia patients. (A) Red cells were pre‐loaded with CM‐H_2_DCF‐DA (100 μmol/l) or treated with the same final [DMSO] before incubation with HOCl (500 μmol/l) at 0·5% haematocrit for 30 min at 37°C in HK‐HBS (*n* = 3). (B) Red cells were permeabilised to Ca^2+^ as in Fig 1B at 0·5% haematocrit for 30 min at 37°C in the absence (full circles) or presence (open circles) of HOCl (500 μmol/l) after which accessible PS was labelled with LA‐FITC (*n* = 6). PS exposure was normalised to that of control red cells at 1 μmol/l free [Ca^2+^]_o_ (32 ± 5·4% of total red cells). Symbols represent means ± SEM for red cells from *n* different individuals.

The final oxidative manoeuvre tested was exposure HOCl. This oxidant is produced by myeloperoxidase, released from activated neutrophils, which are important mediators of vascular inflammation in SCA. Like NEM, HOCl may also react with membrane thiols. At higher concentrations of HOCl, the red cell oxidant load measured using CMH_2_DCF achieved a similar level to that obtained with other oxidants although no plateau was observed (Fig 5A). The effect of HOCl on PS exposure measured using LA‐FITC was tested at concentrations up to 500 μmol/l, however, no effect was observed when compared with untreated red cells (Fig 5B).

## Discussion

Surprisingly, the present findings show that a number of oxidants – xanthine oxidase/hypoxanthine (XO/HO) mixture, PMS, NO_2_ and *t*BHP – inhibited Ca^2+^‐induced PS exposure in red cells from patients with SCA, with little effect on its Ca^2+^ dependence. In contrast, thiol oxidation with NEM markedly stimulated lipid scrambling with increase in Ca^2+^ affinity, whilst protection of reduced thiols with DTT inhibited PS exposure. HOCl, however, was without effect.

Red cell PS exposure is observed in numerous pathological conditions, including SCA, in which a significant, but variable (2–10%), percentage of circulating red cells are positive for externalised PS (Wood *et al*, 1996; Kuypers, 1998; de Jong *et al*, 2001; Dasgupta & Thiagarajan, 2005; Cytlak *et al*, 2013). SCA is associated with increased oxidative stress, neutrophil leucocytosis and vascular endothelial dysfunction, all of which are also associated with increased PS exposure (Jain, 1985; Hebbel, 1991; Kuypers, 1998; Mutze *et al*, 2003; Banerjee & Kuypers, 2004; Zhang *et al*, 2013). Recent reviews have reinforced the view that ROS are actively involved in lipid scrambling in SCA (Cimen, 2008; Mohanty *et al*, 2014; Voskou *et al*, 2015), with limited experimental evidence supporting this assumption. Aside from thiol modification with NEM (de Jong & Kuypers, 2006), however, definitive evidence for stimulation of PS exposure by oxidant challenge is scant.

**Figure 6:**
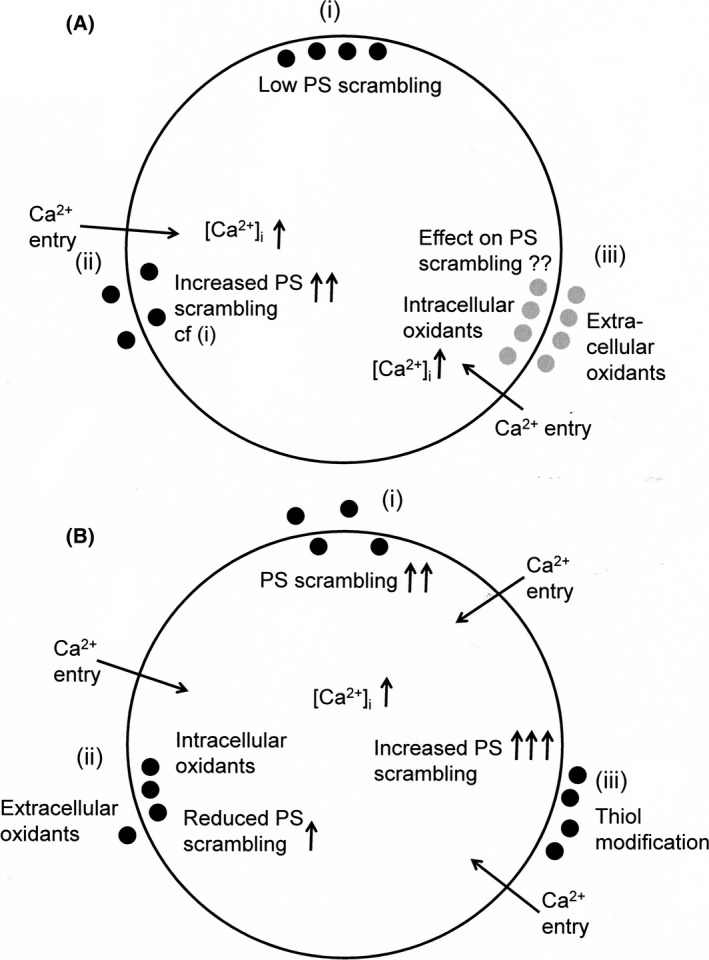
Schematic digram of stimuli affecting phosphatidylserine (PS) distribution in red cells from patients with sickle cell anaemia. (A) (i) PS is usually confined to the inner leaflet of the lipid bilayer of red cells including sickle cells through high activity of the flippase and low activity of the scramblase, as externalisation is prothrombotic and increases phagocytosis; (ii) elevation of intracellular Ca^2+^ ([Ca^2+^]_i_) via the deoxygenation‐induced cation conductance (or P_sickle_) or via ionophore promotes PS exposure increasing the possibility of microvascular occlusion; (iii) the effect of oxidants either from within the sickle cell or from the circulation is uncertain. (B) (i) As before, entry of Ca^2+^ increases PS exposure; (ii) most oxidants (xanthine oxidase/hypoxanthine mixtures, nitrite, phenazine methosulphate) actually reduced Ca^2+^‐induced PS exposure by about 50% and would reduce thrombosis; (iii) the exception was thiol oxidation which markedly increased externalisation of PS. ●, known PS distribution; 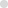, PS distribution unknown.

We used four different oxidant treatments (XO/HO mixtures, PMS, NO_2_ and *t*BHP) to investigate their effects on PS exposure in red cells from SCA patients. These particular oxidants were chosen because of the qualitative differences in oxidative challenge that they present to the red cell. XO/HO mixtures generate SOA and hydrogen peroxide extracellularly (Baskurt *et al*, 1998; Rogers *et al*, 2009), whilst PMS generates SOA intracellularly (Nishikimi *et al*, 1972; Maridonneau *et al*, 1983); NO_2_ oxidises Hb to metHb (Muzyamba *et al*, 2000); *t*BHP increases generation of peroxyl and alkoxyl free radicals (Davies, 1989); whilst HOCl produced by myeloperoxidase released from neutorphils may also oxidise membrane thiols (Vissers *et al*, 1994; Gorudko *et al*, 2016). Red cell PS exposure is most reliably stimulated by elevation of intracellular Ca^2+^ (Bevers & Williamson, 2010) whilst a number of oxidants have previously been shown to increase red cell cation permeability (Gibson & Muzyamba, 2003a,2003b). We therefore hypothesised that oxidants may act synergistically with intracellular Ca^2+^ to increase PS exposure and alter its Ca^2+^ affinity, thereby accounting for the raised percentage of PS‐positive red cells in SCA patients. Under the experimental conditions used, however, instead of stimulating PS exposure, we found that these oxidants produced significant inhibitory effects on PS exposure (by about 40–60%) with no apparent change in Ca^2+^ dependence. These findings are summarised in Fig 6.

Oxidants are also associated with PS shedding and microvesiculation (Piccin *et al*, 2007, 2015b; Freikman *et al*, 2008; Alaarg *et al*, 2013) and it was possible that reduced PS levels on the outer bilayer of the red cell membrane could be due to its loss into the incubation media, notwithstanding an increase in lipid scrambling. PS may be lost as microvesicles, which have been proposed as a biomarker for SCA severity (Piccin *et al*, 2015a). Total thrombin formation in permeabilised red cells (using hypotonic lysis) were similar in controls and after oxidant challenge, however, which makes it unlikely that large reductions in labelled PS on the outer bilayer (up to 65%) could result from PS shedding. Microvesicles may, however, be formed in response to Ca^2+^‐induced PS scrambling. The mechanisms by which oxidants decrease PS exposure are unclear, although membrane lipid and protein damage, for example by peroxidation, may specifically inhibit the scrambling protein transporter. Conversely, lipid peroxidation may reduce the ability of the scramblase to translocate PS through the lipid bilayer.

The one manoeuvre which did increase PS exposure markedly was incubation with NEM to cause thiol oxidation (Fig 6). Thiol reactions are known affect the activities of the flippase and scramblase (Connor & Schroit, 1990; Martin & Jesty, 1995; Kamp *et al*, 2001). NEM has previously been shown to increase scrambling in normal human and mice red cells (Kamp *et al*, 2001; de Jong & Kuypers, 2006) and our experiments confirm this effect in red cells from SCA patients. An important difference with previous work, however, is that we observe marked effects on PS exposure at two orders of magnitude lower Ca^2+^ concentrations. PS exposure began with elevation of red cell Ca^2+^ concentration to about 100–200 nmol/l. In the opposite way, reduction of thiols by DTT reduced scrambling, as seen previously for pyridyldithioethylamine (de Jong & Kuypers, 2006). Alterations in Ca^2+^ affinity in our hands were modest but there was a slight increase in the presence of NEM, in contrast to previous work (de Jong & Kuypers, 2006) (cf. de Jong *et al*, 1997) but note the difference in haematocrit). The action of DTT *in vitro* suggests that circulating sickle cells are already deprived of reduced thiols (Kamp *et al*, 2001). Indeed, sickle cells are reported to contain lower levels of membrane thiols (Rank *et al*, 1985; Rice‐Evans *et al*, 1986). HOCl also reacts with membrane thiols. However, although giving a similar level of oxidant challenge to the red cell, as assessed using CMH_2_DCF, HOCl had no effect on Ca^2+^‐induced PS exposure. The lack of effect of HOCl suggests that NEM is able to gain access to key targets which are unavailable to HOCl. Nevertheless, it is likely that thiol modification accounts for the greater percentage of circulating sickle cells positive for PS.

**Figure 7:**
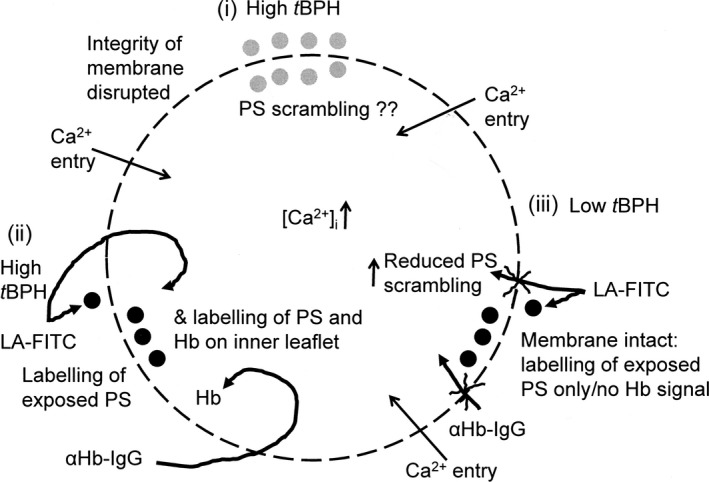
Schematic diagram of the effect of *tert*‐butyl hydroperoxide (*t*BHP) on red cells from patients with sickle cell anaemia. (i) *t*BHP is thought to increase phosphatidylserine (PS) exposure but relatively high concentrations have been used, which may disrupt the integrity of the membrane; (ii) in the presence of high *t*BHP more PS was labelled, but the ability of anti‐Hb IgG to label intracellular Hb suggests that this was because loss of membrane integrity allowed labelling of both inner leaflet PS as well as externalised PS; (iii) by contrast, with lower *t*BHP concentrations that do not allow entry of anti‐Hb IgG, PS exposure was reduced by 50%, as for the other oxidants (in Fig 1b). ●, known PS distribution; 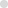, PS distribution unknown.

Finally, red cells from SCA patients have been previously described to show increased PS exposure on incubation with *t*BHP (Lang *et al*, 2002). The concentrations used in this previous report were up to 1 mmol/l, however, with an incubation time of 2 h, after which all red cells were positive for PS (labelled using FITC‐ annexin V). Our findings that red cells treated with similar concentrations of *t*BHP become positive to fluorescently‐labelled antibodies to Hb α chain within 30 min suggest that membrane disintegrity occurs at these higher concentrations and that the PS label can gain access to cytoplasmic PS as well as externalised PS (Fig 7). This hypothesis is supported by previous observations that large cation leaks are induced in red cells at higher [*t*BHP] (Ney *et al*, 1990).

In conclusion, the experiments described in the present work show that a number of different oxidants (XO/HO mixtures, PMS, NO_2_ and *t*BHP) reduced Ca^2+^‐induced PS exposure in red cells from SCA patients. Thiol oxidation by NEM was confirmed to increase PS exposure, whilst thiol reduction using DTT decreased it, consistent with the postulate that thiol oxidation *in vivo* contributes to increased numbers of PS‐positive red cells in SCA patients. These findings are particularly relevant to the ability of oxidative stress in the circulation *in vivo* in SCA patients to participate in thrombus formation, vascular occlusion and tissue ischaemia via mechanisms involving red cell PS, and demonstrate the complexity of the pathophysiology of sickle cell disease. This study gives important new information on the complex role of oxidative stress in the pathophysiology of sickle cell disease and suggests that the effects may vary depending on the precise nature of the oxidants and their physiological context.

## Conflicts of interest

There are no conflicts of interest.

## Author contributions

Most experiments were carried out by AH with some assistance from AN and BL; DCR and JSG designed the study; JNB and DCR acquired samples; AH and AN analysed the data; JSG, DCR, JNB and AH prepared the manuscript.

## Supporting information


**Appendix S1.** Materials and methods.
