# Supplementary material for: Oxidative stress and phosphatidylserine exposure in red cells from patients with sickle cell anaemia
Source: Br J Haematol. 2018 Jun 25;182(4):567–78. doi: 10.1111/bjh.15441 (PMC6120535; doi:10.1111/bjh.15441)
Supplement: Supplementary file 1 — Appendix S1. Materials and methods. [file BJH-182-567-s001.doc]

**Materials and Methods**

*Chemicals*

Fluorescein isothiocyanate-conjugated lactadherin (LA-FITC), human prothrombin, human factor Va and human factor Xa came from Haematologic Technologies Inc. (Essex Junction, VT, USA), supplied by Cambridge Bioscience (Cambridge, UK). Alexa Fluor 647 conjugated anti-haemoglobin subunit α immunoglobulin came from Abcam (Cambridge, UK), anti-glycophorin A-phycoerythrin (PE) from Becton Dickinson Biosciences (CA, USA), and ThrombinChrom from Enzyme Research Laboratories (Swansea, UK). Bromo-A23187, 5-(and-6)-chloromethyl-2',7'-dichloro-dihydro-fluorescein diacetate acetyl ester (CM-H_2_DCF-DA) and 4-(2-hydroxyethyl)-1-piperazineethanesulfonic acid (HEPES) came from Calbiochem (Merck, Darmstadt, Germany). All other chemicals were supplied by Sigma-Aldrich Co. (Poole, Dorset, UK).

*Sample collection and handling*

Consented blood samples were taken from children homozygous for sickle cell anaemia (SCA), HbSS genotype, into the anticoagulant EDTA. The study was approved by the National Research Ethics Committee (reference 16/LO/1309). For some experiments, once routine haematological assays had been completed, discarded and anonymised blood was used. All research was conducted with ethical approval and in accordance with the Helsinki Declaration of 1975, as revised in 2008. The study involved thirty seven patients, twenty three of whom were male and fourteen female. Average age was 11.3 ± 3.8 years. Nineteen were receiving hydroxyurea therapy and eighteen were not. Mean % HbF levels and reticulocyte counts were 10.2 ± 5.1 and 13.2 ± 5.1, respectively (all means ± S.D., n = 37).

*Solutions and red cell preparation*

The standard saline was one containing high K^+^ levels (HK-HBS), buffered with HEPES, and comprised (in mM): NaCl 54, KCl 90, MgCl_2_ 0.15, inosine 10 and HEPES 10, (pH 7.4 at 37 °C; 290 ± 5 mOsm.kg^-1^). For Ca^2+^ titration curves, additions of EGTA (2 mM) and concentrations of total [Ca^2+^] of 0, 1.35, 1.63, 1.72, 1.81, 1.85, 1.91 and 2.00 mM were used to clamp free extracellular [Ca^2+^] ([Ca^2+^]_o_) at 0, 0.1, 0.2, 0.3, 0.45, 0.6, 1 and 10 µM, respectively. When xanthine oxidase (XO) was used, buffers also contained 2mM hypoxanthine (HO). Where required, red cells were permeabilised to Ca^2+^ with the ionophore bromo-A23187 (6 µM final). For oxidant challenge, stock solutions (100x final concentration) of XO, phenazine methosulphate (PMS), *tert*-butyl hydroperoxide (*t*BHP) and sodium nitrite (NO_2_) were made in HK-HBS. To expose cells to hypochlorous acid, NaOCl was diluted with HK-HBS immediately before use and the pH then readjusted to 7.4. At this pH the solution will contain approximately 1:1 HOCl and OCl^-^, and is subsequently referred to as HOCl (Vissers, Stern et al. 1994). CM-H_2_DCF-DA was used as a measure of red cell oxidant challenge and was dissolved in DMSO. In experiments using this fluorophore, control red cells were also treated with the same [DMSO] (≤ 0.5 % final). PS levels were measured using LA-FITC or prothrombinase assays. Red cells were labelled with LA-FITC (16 nM) in HK-HBS (pH 7.4 at room temperature, RT) containing 1 mM vanadate (LA-FITC binding buffer). For measurement of prothrombinase activity, the basic saline comprised (in mM): NaCl 54, KCl 90 and tris (hydroxymethyl)aminomethane (Tris) 10 (HK-TBS). Prothrombinase incubation medium comprised HK-TBS with additions of CaCl_2_ (4 mM) and human serum albumin (HSA, 0.5 mg.ml^-1^), pH 7.8 at RT (HK-Tris / Ca^2+^ / HSA). The stop medium was HK-TBS with additional EDTA (2 mM), pH 7.5 at RT (HK-TBS / EDTA). ThrombinChrom (4 mM) was dissolved in water. To prepare red cells, whole blood was washed four times in HK-HBS (pH 7.4 at RT) to remove plasma and buffy coat. For experiments in which intracellular [Ca^2+^] ([Ca^2+^]_i_) was manipulated, the final two washes contained, in addition, EGTA (1 mM) to remove any contaminant Ca^2+^. Red cells were stored on ice until required. Haematocrit (Hct) was measured using Drabkin's reagent.

*Oxidant challenge and thiol modifications*

Extracellular superoxide anion (SOA) and hydrogen peroxide was generated by incubation with mixtures of HO (2 mM) and XO at concentrations up to 0.1 units.ml^-1^, a manoeuvre which is well established to provide an oxidative challenge to red cells (Baskurt, Temiz et al. 1998; Rogers, Said et al. 2009). Using data from pilot experiments, most work was carried out with a [XO] of 0.015 units.ml^-1^. PMS (0.01 – 0.4 mM) was used to generate intracellular superoxide anion. NO_2_ (to generate methaemoglobin) and *t*BHP (to generate peroxyl and alkoxyl derivatives) were used at concentrations of 1 – 20 mM and 0.05 – 1.0 mM, respectively. HOCl was prepared immediately before experiments and used at a final concentration of 0.001 – 1 mM. Red cells were incubated with oxidants for 30 min at 37 ^o^C at an Hct of 0.5 % prior to measuring PS. The thiol modifiers *N*-ethylmaleimide (NEM) and dithiothreitol (DTT) were used at concentrations of 1 mM and 0.25 mM and red cells pre-incubated for 30 min at 37 ^o^C and an Hct of 4 %, followed by 30 min at 37 ^o^C at an Hct of 0.5 % without or with oxidants. DTT was excluded from the second incubation step to prevent red cell lysis.

*Measurement of red cell oxidative stress and red cell morphology*

To measure intracellular oxidative load, washed red cells (in HK-HBS, pH 7.4) were first loaded with CM-H_2_DCF-DA (100 µM) in the dark (10 % Hct for 30 min at 37 ^o^C), washed twice and resuspended in HK-HBS. On permeation into red cells, this fluorophore is hydrolyzed to the non-fluorescent di-hydro compound which, in the presence of reactive oxygen species (ROS), is oxidized to highly fluorescent CM-H_2_dichlorofluorescein (CM-H_2_-DCF). After incubation in the dark with the various oxidants (at 0.5 % Hct for 30 min at 37 °C), red cells were pelleted and the supernatant removed. Red cells were then resuspended at a final Hct of 0.01 % and kept on ice in the dark until CM-H_2_DCF fluorescence was measured in the FL1 channel of a BD Accuri C6 flow cytometer (Becton Dickinson, Oxford, UK), with excitation and emission wavelengths of 488 nm and 530 nm, respectively. Median FL1 fluorescence was used as an indication of the magnitude of intracellular oxidative load. Forward scatter (FSC, cell size) and side scatter (SSC, cell granularity) gates for red cells were identified in control experiments using anti-glycophorin A-PE labelled red cells (Cytlak, Hannemann et al. 2013). For each fluorescence measurement 10,000 events were gated. To examine red cell morphology, cells were fixed through addition of glutaraldehyde (0.3 %) and examined under light microscopy, typically examining several hundred cells.

*Measurement of externalised PS using LA-FITC*

To promote lipid scrambling, red cells were incubated in HK-HBS (0.5 % Hct; 30 min, 37 °C) containing 2 mM EGTA at various [Ca^2+^]_o_s, using bromo-A23187 (6 µM) to permeabilise red cells to Ca^2+^, in the absence or presence of different oxidants or thiol modifiers. The activity of bromo-A23187 was abrogated by adding 0.4 mM Co^2+^. Red cells were then pelleted and resuspended in HK-HBS containing 1 mM vanadate (pH 7.4 at RT), diluted in LA-FITC binding buffer at 0.01 % Hct, and incubated for 15 min in the dark at RT. Red cells were next pelleted (10 s at 16,100 g), washed once in HK-HBS, resuspended and kept on ice in the dark until flow cytometry analysis. LA-FITC was detected in the FL1 channel of a BD Accuri C6 flow cytometer using logarithmic gain (as for CM-H_2_-DCF fluorescence). The positive fluorescent gate was set using red cells unlabelled with LA-FITC. For each measurement 10,000 events were gated. PS positive cells were defined as all events falling within the preset FSC, SSC and positive fluorescent gates. XO, PMS and *t*BHP all showed various degrees of autofluorescence in unlabelled red cells (in the absence of any fluorophore). It was therefore critical to choose appropriate concentrations of these oxidants which elevated intracellular ROS levels whilst keeping autofluorescence to manageable values. In all cases, compensation for the fluorescent overspill was carefully set using oxidant-treated red cells, unlabelled with LA-FITC. NO_2_ showed no significant autofluorescence in the FL-1 channel.

*Measurement of externalised PS using a prothrombinase assay*

PS exposure was also assessed by generating thrombin using a prothrombinase assay, following the method of Bevers *et al.* 1982 (Bevers, Comfuriius et al. 1982). Red cells were incubated in the absence or presence of HO (2 mM) / XO (0.015 units.ml^-1^) mixtures or PMS (0.1 mM), together with various [Ca^2+^]_o_s and bromo-A23187. Ca^2+^ permeabilisation was then stopped with 0.4 mM Co^2+^ and bromo-A23187 was removed by three washes in HK-HBS containing 10 mg.ml^-1^ BSA, followed by one wash in HK-TBS, before cells were resuspended at 1 % Hct in HK-TBS / Ca^2+^ / HSA. 2.5 µl of the red cell suspension were added to 57.5 µl HK-TBS / Ca^2+^ / HSA and incubated in a water bath at 37 °C. After 2 min, 20 µl of HK-TBS / Ca^2+^ / HSA containing Factor Va and Factor Xa (0.5 units.ml^-1^ and 0.25 units.ml^-1^ final concentration, respectively) were added, and after a further 2 min thrombin formation was initiated by adding 20 µl of HK-TBS / Ca^2+^ / HSA containing prothrombin (1.1 µM final concentration). After 2 and 5 min, 5 µl aliquots were removed and 150 µl of HK-TBS / EDTA added to stop thrombin formation. Thrombin levels were measured by adding the chromogenic substrate ThrombinChrom (0.125 mM final concentration) with absorbance measured at 415 nm in 96-well plates using an iMark microplate reader (Bio-Rad, Hemel Hempstead, UK). Change in absorbance with time was used to calculate the relative amount of thrombin formed per minute, which provides an indirect measure of the presence of externalised PS. Further aliquots of untreated red cells were also lysed hypotonically through addition of water (0.25 % Hct), following which the prothrombinase assay was repeated thus giving a measure of total red cell PS in both the inner and outer bilayers of the membrane. The relative percentage of exposed PS to total red cell PS was then calculated. All results were corrected for Hct. Although more complicated to carry out, this prothrombinase assay has the advantage of being immune to any problems with autofluorescence or setting of positive gates in the flow cytometer.

*Measurement of red cell membrane integrity*

Red cells were treated with *t*BHP (0 – 1 mM) in HK-HBS (0.5 % Hct, pH 7.4 for 30 min at 37 °C), washed and resuspended in HK-HBS. Four aliquots were prepared: (i) CM-H_2_DCF-labelled only, (ii) LA-FITC-labelled only, (iii) dual labelled with CM-H_2_DCF and Alexa Fluor 647 conjugated anti-Hb subunit α immunoglobulin (1:100 dilution) and (iv) dual labelled with LA-FITC and Alexa Fluor 647 conjugated anti-Hb subunit α immunoglobulin (1:100 dilution). CM-H_2_DCF-DA and Alexa Fluor 647 anti-Hb were added to red cells at 0.01 % Hct in HK-HBS, for 30 min at RT in the dark. LA-FITC (16 nM) was added for the final 15 min. Unlabelled controls were treated with DMSO at the same concentration as present with the fluorophores. Red cells were pelleted (10 s at 16,100 g), washed once in HK-HBS, resuspended and kept on ice in the dark until flow cytometry analysis. Median CM-H_2_DCF and LA-FITC fluorescence were detected as described above. Median fluorescence of Alexa Fluor 647 conjugated anti-Hb was detected in the FL4 channel of a BD Accuri C6 flow cytometer (excitation 640 nm; emission 675 nm). Immunoglobin (molecular weight 15 kDa) cannot cross the red cell membrane unless its permeability barrier has been compromised, therefore an increase in median FL4 signal was taken as an indication of loss of membrane integrity (Figure 3). If this was the case, LA-FITC would also be expected to gain access to the red cell interior (as well as the exterior) and therefore label PS in both the inner, as well as the outer, bilayer of the membrane. Positive LA-FITC red cells would therefore not be restricted to those with only externalised PS.

*Statistics*

Results are presented as means ± S.D. or S.E.M. for blood samples from *n* different individuals. Where appropriate, comparisons were made using 2-tailed Student's *t*-tests and p < 0.05 was considered as significant.
